# A 3 months follow-up of functional dependency, muscle strength, balance and endurance in long-stay icu patients; association with rehabilitation programmes

**DOI:** 10.1186/2197-425X-3-S1-A442

**Published:** 2015-10-01

**Authors:** CM de Jager, A van Wieren, NA Bruins, EC Boerma

**Affiliations:** Medical Centre Leeuwarden, Intensive Care Medicine, Leeuwarden, the Netherlands

## Introduction

Over the last decades ICU survival is increasing. As a consequence, post ICU sequelae with (persistent) functional dependency in long-stay ICU patients become a topic of interest. However data on specific fields of physical performance and the potential influence of rehabilitation programmes remain scarce [1].

## Objectives

To establish the course of functional dependency and specific fields of physical performance in patients with and without a (non ICU-specific) rehabilitation programme in long-stay ICU patients.

## Methods

We performed a single centre prospective observational study in mixed ICU patients with a length of stay ICU > 48 hours. Patients were evaluated at ICU discharge and re-evaluated after a 3-months period in a specific post-ICU outpatient clinic. At both time points a Barthel score (BS) and grip strength (GS) were recorded. Additionally the 6 minutes walking test (6MWT), the Berg balance scale (BBS) and characteristics of individual rehabilitation programmes were assessed in the outpatient setting. Non-parametric tests for comparison between dependent and independent datasets were used. A Bonferroni correction was used for multiple comparison.

## Results

In a 1-year period during 2014 80 patients were included. Baseline characteristics are provided in Table 1. Overall there was a statistical improvement over time in all functional fields (Table 2). At 3 month 5 patients (6%) remained fully/severely functionally dependent, 3 (4%) were partially functionally dependent, 16 (20%) became functionally independent with help and 56 (70%) became functionally independent. At this time point grip strength returned to normal in at least 1 hand in 90%, and in both hands in 81% of patients. The BBS at three months indicated a low fall risk in 97% of patients. However, the 6MWT at three months was only normalized in 19% of patients. 52% of patients participated in any form of (non ICU-specific) rehabilitation program, 48% did not take part in any form of rehabilitation programme. There was no statistical difference in any outcome variable of physical performance between patients with and without a rehabilitation program.

**Table 1:** Baseline characteristics.

	All (N = 80)	Non-rehab (N = 36)	Rehab (N = 39)	p-value
Age (years)	65[56-73]	65[57-73]	65[56-72]	0.89
APACHE II score	22[16-30]	21[15-24]	29[24-33]	0.001
Admission type				
-elective(%)	16	17	15	0.88
-non-elective(%)	84	83	85	
Ventilator days	4[3-9]	4[2-6]	5[3-10]	0.044
LOS ICU (days)	23[15-35]	20[13-37]	27[19-34]	0.33
Data are presented as median [IQR]. APACHE Acute physiology and chronic health evaluation, LOS Length of stay, Rehab Rehabilitation programme. P value between subgroups (rehab versus non-rehab)

**Table 2:** Results.

	ICU discharge	3 Month all	Without rehab	With rehab	p-value
Barthel score, AU	12[8-16]	20[18-20]	20[20-20]	20[18-20]	^a^0.006 ^b^0.84
Grip strength L, KPa, (%)	18[14-23],(36)	29[23-40], (90)	32[26-40]	26[21-37]	^a^0.006 ^b^0.90
Grip strength R, KPa, (%)	19[14-29], (28)	34[24-44], (84)	38[27-46]	28[22-39]	^a^0.006 ^b^0.16
6MWT, m, (%)	NA	420[350-550], 86[68-93]	460[360-554], 87[73-93]	395[338-538], 84[63-93]	^b^1.0
Berg Balance Scale, AU	NA	54[52-56]	54[52-56]	54[50-56]	^b^1.0
Data are presented as median [IQR] or in percentage of normal values. 6 MWT 6 minutes walking test, Rehab Rehabilitation programme.^a^ comparison between ICU discharge and 3 months after ICU discharge, ^b^ comparison between subgroups with and without rehabilitation programme					

## Conclusions

In long-stay ICU patients overall physical performance considerably improved in 3 months from ICU discharge. However, there are large differences in the recovery of specific fields of physical performance, irrespective of participation in non ICU-specific rehabilitation programmes. Further research to unravel the specific rehabilitation needs of long-stay ICU patients is needed.
